# Insulin-Sensitizers, Polycystic Ovary Syndrome and Gynaecological Cancer Risk

**DOI:** 10.1155/2016/8671762

**Published:** 2016-09-20

**Authors:** Rosa Lauretta, Giulia Lanzolla, Patrizia Vici, Luciano Mariani, Costanzo Moretti, Marialuisa Appetecchia

**Affiliations:** ^1^Unit of Endocrinology, Regina Elena National Cancer Institute, Rome, Italy; ^2^Unit of Endocrinology, Department of Systems' Medicine, University of Rome Tor Vergata, Section of Reproductive Endocrinology, Fatebenefratelli Hospital “San Giovanni Calibita” Rome, Italy; ^3^Division of Medical Oncology B, Regina Elena National Cancer Institute, Rome, Italy; ^4^Department of Gynaecologic Oncology, HPV-Unit, Regina Elena National Cancer Institute, Rome, Italy

## Abstract

Preclinical, early phase clinical trials and epidemiological evidence support the potential role of insulin-sensitizers in cancer prevention and treatment. Insulin-sensitizers improve the metabolic and hormonal profile in PCOS patients and may also act as anticancer agents, especially in cancers associated with hyperinsulinemia and oestrogen dependent cancers. Several lines of evidence support the protection against cancer exerted by dietary inositol, in particular inositol hexaphosphate. Metformin, thiazolidinediones, and myoinositol postreceptor signaling may exhibit direct inhibitory effects on cancer cell growth. AMPK, the main molecular target of metformin, is emerging as a target for cancer prevention and treatment. PCOS may be correlated to an increased risk for developing ovarian and endometrial cancer (up to threefold). Several studies have demonstrated an increase in mortality rate from ovarian cancer among overweight/obese PCOS women compared with normal weight women. Long-term use of metformin has been associated with lower rates of ovarian cancer. Considering the evidence supporting a higher risk of gynaecological cancer in PCOS women, we discuss the potential use of insulin-sensitizers as a potential tool for chemoprevention, hypothesizing a possible rationale through which insulin-sensitizers may inhibit tumourigenesis.

## 1. Introduction to Polycystic Ovary Syndrome

**Figure 1:**
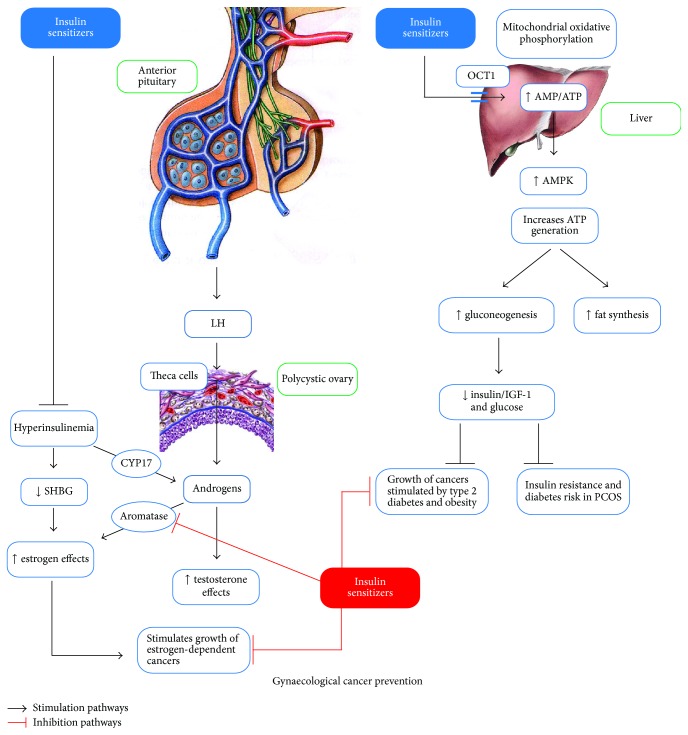
The combined action of insulin-sensitizers on the liver and ovary and the supposed protecting effect on endocrine-related gynaecological cancer. In the liver metformin inhibits mitochondrial respiratory complex 1 promoting AMPK activation which improves the metabolic profile reducing hyperglycemia, hyperinsulinemia, and insulin resistance. Insulin-sensitizers have a positive effect in PCOS patients through normalization of hyperinsulinaemia that otherwise amplifies the excessive androgen production from the ovary theca cells via CYP17 phosphorylation and lower levels of SHBG. Metformin and inositols may play an anticancer role both as insulin-sensitizers and as aromatase inhibitors. Indeed, they improve metabolic profile, inhibiting the growth of tumoural cells stimulated by hyperinsulinemia, and normalize estrogen production, inhibiting the growth of estrogen-dependent cancers. OCT1: Organic Cation Transporter 1; LH: luteinizing hormone; ISF-1: insulin sensitivity factor; CYP17: Cytochrome P-45017; SHBG: sex hormone binding globulin; IGF-1: insulin-like growth factor; PCOS: polycystic ovary syndrome.

Polycystic ovary syndrome (PCOS) is one of the most common endocrine disorders in women of reproductive age, with an estimated incidence rate of 5–10% [1, 2]. The syndrome has a heterogeneous presentation, which includes hirsutism, often related to hypersecretion of ovarian androgens, anovulation, menstrual irregularity, infertility, and pregnancy complications. PCOS may predispose women to cardiovascular and metabolic dysfunction as well as an increased risk of type 2 diabetes [3]. The excess of ovarian androgen secretion [4] may affect raised pituitary luteinizing hormone (LH) production and in addition contributes to the mechanisms of anovulation. Insulin sensitivity and secretion can be affected by hyperandrogenism; however dietary factors and independent genetic seem to have also a role [5]. Hyperinsulinemia and peripheral insulin resistance are present in about half of PCOS women mainly in adipose tissue and skeletal muscle while ovarian theca and granulosa cells have been reported to be highly sensitive to insulin. Insulin stimulates ovarian theca cells to produce androgen (i.e., testosterone) through the stimulation of the insulin receptor (IR) like LH [6]. Both hypersecretion of LH and hyperinsulinemia cooperate to increase ovarian theca cell androgen production contributing to androgen dependent hirsutism also by suppression of hepatic secretion of sex hormone binding globulin (SHBG), which increases the bioavailability of circulating testosterone [7]. The use of antihyperglycemic drugs enhancing peripheral insulin sensitivity is widely used to treat metabolic aspects in PCOS women often from a long time [8]. However, the correction of hyperinsulinemia leads to a decreased ovarian androgen production. Chan indicates that using insulin-sensitizers may have a role as a tool for cancer prevention [9]. In the present review we try to hypothesize a possible rationale through which insulin-sensitizers may inhibit tumourigenesis (Figure 1).

## 2. Insulin Receptor Signaling and Phosphoinositide Pathways

**Figure 2:**
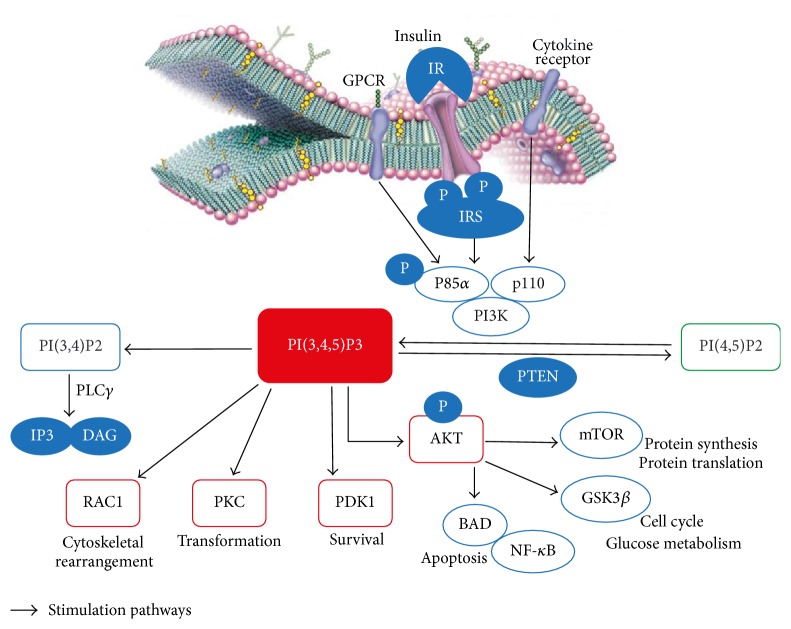
Insulin receptor and phosphatidylinositol (3,4,5)-trisphosphate formation. The activation of PI3K by insulin, G-protein coupled receptor, or growth factors generates different 3′ phosphorylated inositol products which in turn recruit effector molecules regulating many cellular functions involved in cells survival and metabolism. GPCR: G-protein coupled receptor; IR: insulin receptor; IRS: insulin receptor substrate; PI3K: phosphatidylinositol 3-kinase; PI(4,5)P2: phosphatidylinositol 4,5-bisphosphate; PI(3,4,5)P3: phosphatidylinositol 3,4,5-triphosphate; PI(3,4)P2: phosphatidylinositol 3,4-bisphosphate; PLC*γ*: Phospholipase C *γ*; IP3: inositol trisphosphate; DAG: diacylglycerol; PKC: protein kinase C; PTEN: phosphatase and tensin homolog; Akt: protein kinase B; RAC1: Ras-Related C3 Botulinum Toxin Substrate 1; PDK1: phosphoinositide dependent protein kinase-1; mTOR: mammalian target of rapamycin; GSK3*β*: glycogen synthase kinase 3; NF-*κ*B: nuclear factor *κ*B; BAD: Bcl2-Antagonist of cell Death.

Insulin receptor is a transmembrane receptor encoded by a single gene, belonging to the large class of tyrosine kinase receptors [3]. It is activated by insulin, insulin growth factor 1 (IGF-I), and insulin growth factor 2 (IGF-II) [16]. The main activity of the IR when bound by an insulin molecule is inducing glucose uptake. For this reason a decreased sensitivity in insulin receptor signaling, associated with impaired glycogen synthesis and inhibition of glycogen breakdown, progressively leads to metabolic disorders and type 2 diabetes mellitus [17]. Peripheral insulin resistance is then defined by a decrease in insulin-dependent glucose transport at the level of target tissues [18] due to defects at both the insulin receptor and/or postreceptor signaling [19]. Following hormone binding the IR undergoes conformational changes which allow autophosphorylation of its tyrosine residues, docking sites for insulin receptor substrates (IRSs) involved in phosphatidylinositol-3-kinase (PI3K) activation and recruitment to the plasma membrane of the serine/threonine protein kinase Akt/PKB which represents the main intracellular interconnecting pathway activated to ensure insulin biological action together with the mitogen-activated protein kinase (MAPK)/extracellular-signaling regulated protein kinase 1/2 (ERK 1/2) pathway [20, 21]. In mammals, almost five isoforms of the regulatory subunit of PI3K interact with IRSs activating the catalytic subunit and phosphorylating the phosphatidylinositol 4,5-biphosphate [PI(4,5)P2] which in turn enables other phosphoinositide dependent kinases [mainly phosphatidylinositol 3,4,5-trisphosphate, PI(3,4,5)P3] involved in the phosphoinositide signaling system and in the signaling of the glucose transporter type 4 (GLUT4) the main GLUT-like protein identified in mammals [22]. The phosphoinositide signaling system plays a pivotal role in the regulation of cellular processes, such as vesicle transport cell proliferation and cytoskeletal remodeling [23] and its deregulation may lead to many diseases including cancer [24]. Glucose uptake for cellular function is additionally regulated by a sensor of intracellular energy level, the 5′ adenosine monophosphate-activated protein kinase (AMPK) pathway. AMPK is a highly conserved serine/threonine protein kinase that appears to universally exist as heterotrimeric complex comprised of catalytic *α*-subunit and regulatory *β*- and *γ*-subunits [25]. Like several other protein kinases, AMPK and its orthologues are only significantly active when phosphorylated by upstream kinases at the level of a conserved threonine (Thr172) within the kinase domain [26]. In mammals, the main upstream kinase phosphorylating Thr172 is the liver kinase 1-Ste20-Related Adaptor-Mouse protein 25 (LKB1-STRAD-MO25) heterotrimeric complex, a biologically active unit containing the tumour suppressor kinase LKB1 [27, 28]. This complex is constitutively active and presents a high basal activity [29]; however its ability to phosphorylate Thr172 is improved by conformational changes in its AMPK substrate, due to the binding of 5′ adenosine monophosphate (AMP) active to its *γ*-subunit. Moreover, kinases that can activate AMPK also include calcium/calmodulin-dependent protein kinase (CaMKK) [30] and transforming growth factor *β*-activated kinase 1 (TAK1) [31]. Overall, AMPK maintains cellular energy homeostasis and its activation by metabolic stress (accountable of energy depletion) leads to inhibition of cell growth and promotion of adenosine-5′-triphosphate (ATP) production. Indeed, once activated, AMPK can encourage catabolic pathways and inhibit anabolic pathways to restore energy stores [32]. Furthermore, many of the biosynthetic pathways, such as synthesis of triglycerides, fatty acids, phospholipids, sterols, glycogen, proteins, and ribosomal RNA, are switched off by AMPK. These pathways are subject to a AMPK dependent downregulation via multiple mechanisms, involving both phosphorylation of pathway key enzymes and longer-term effects on gene expression [32]. As a molecular target of metformin, AMPK is a known key point in the treatment of metabolic syndrome and type 2 diabetes. Recently, AMPK is emerging as a possible metabolic tumour suppressor and a target for cancer prevention and treatment. Moreover, it has been observed that AMPK downstream targets can influence many effector proteins involved in various regulatory processes that contribute to the pathogenesis of cancer. Even if AMPK negatively regulates cyclooxygenase 2 (COX-2), a proinflammatory enzyme associated with tumourigenesis, and can induce phosphorylation of tumour suppressor p53, resulting in cell cycle arrest, the main molecular mechanism involved in the AMPK-mediated tumour suppression is the negative regulation of PI3K/Akt/mTOR signaling pathway [33]. mTOR is a serine/threonine protein kinase that regulates cellular processes including proliferation, growth, motility, survival, protein synthesis, and transcription [34, 35]. mTOR forms two functionally distinct complexes, mTORC1 and mTOR2. mTORC1, a central signaling node that integrates signals arising from growth factors, nutrient availability, and energy status [36], promotes cell growth by phosphorylating ribosomal protein S6 kinase 1 (S6K1) and Eukaryotic Translation Initiation Factor 4E-Binding Protein 1 (4E-BP1) [37, 38]. Inhibition of mTORC1 signaling by AMPK occurs via dual pathways. First, AMPK phosphorylates tuberous sclerosis complex-2 (TSC2) [39] which converts the Ras homolog enriched in the brain (RHEB), a GTP-binding protein, activating upstream mTORC1, to its inactive GDP-bound form. In a second time, AMPK phosphorylates a regulatory-associated protein of mTOR (RPTOR or Raptor), a subunit of the mTORC1 complex, inhibiting it [40, 41]. As AMPK is considered the most important molecular effector of metformin, it may function as an important key-regulator of cellular energy homeostasis. Therefore, it is activated in response to a shortage of energy in order to boost cellular catabolic activities [42]. On the contrary, insulin, which is an anabolic hormone released by nutrient intake, induces cell growth and energy storage. It is therefore not surprising that there is an antagonism between these two pathways. AMPK negatively modulates mTOR through the phosphorylation of TSC2 and Raptor. A similar but functionally opposite dual approach is used by the insulin-signaling pathway to activate mTORC1 and thus contrast AMPK effects [36]. It has been demonstrated that the insulin-activated kinase Akt phosphorylates TSC2 at distinct sites from those specific for AMPK, blocking its RHEB-GAP function [36, 43, 44], and also phosphorylates proline-rich Akt substrate of 40 kDa (PRAS40), enhancing its inhibitory effect on the mTORC1 complex [45]. Hence, AMPK and Akt reciprocally control mTOR pathway. As demonstrated in rat model, the insulin-activated kinase Akt has been shown to phosphorylate the *α*1 catalytic subunit of AMPK at Ser485, reducing the rate of subsequent Thr172 phosphorylation and the LKB1 induced activation in cell-free assay, whereas the pretreatment with insulin may blunt Thr172 phosphorylation during ischemia in perfused rat hearts [46]. The signaling relationship between Akt and AMPK is quite complex. Akt positively regulates mTORC1 and negatively regulates AMPK while some conditions activating AMPK may silence Akt signaling suggesting a bidirectional cross-talk between AMPK and Akt, even if the functional consequence in terms of tumour progression is unclear [33]. For instance, this bidirectional feedback mechanism was shown by Choudhury et al. using a number of prostate cancer cell lines [47]. Therefore, activated AMPK may inhibit or promote Akt signaling depending on the cellular microenvironment and the following phenotypic consequences may dependent on the tumour and cellular context [33] (Figure 2).

Translating into clinical practice the current research data discussed above, it is possible to support the evidence that metformin might induce growth-static effect on several cancers, including pancreatic cancer [48], glioma [49], prostate, and colon cancer [50]. The metformin antiproliferative effect may be exerted through the inhibition of the PI3K/Akt/mTOR signaling transduction pathway [35, 51–54]. Finally, AMPK can also regulate p53 [55] and modulates the activity of transcription factors and coregulators that control the cell cycle [56, 57]. Current evidence suggests that AMPK can act as a tumour suppressor by modulating inflammations, contrasting the metabolic changes that occur during tumourigenesis and directly inducing cell cycle arrest [58].

## 3. Insulin-Sensitizers in PCOS and Cancer Prevention

PCOS is associated with insulin resistance [59] and with a certain number of metabolic disorders [60]. Insulin resistance is a dysmetabolic condition in which a greater amount of insulin is required to exert a physiological cellular response. It is characterized by increased secretion of insulin from pancreatic *β*-cells and compensatory hyperinsulinemia. The treatment of insulin resistance and hyperinsulinemia includes the use of insulin-sensitizers (metformin, thiazolidinediones, and inositols) [8].

### 3.1. Metformin

Metformin is a synthetically derived biguanide, off-label used in the management of dysmetabolic disorders and insulin resistance in PCOS. It has been demonstrated that metformin, when used in addition to changes in the life-style, may restore ovulation in women with PCOS and reduces the risk of ovarian hyperstimulation syndrome [8]. The effects of metformin on glucose metabolism seem to be secondary to its actions on the mitochondrial respiratory chain, inhibiting the mitochondrial respiratory complex I [61, 62]. Therefore, it leads to a reduction in ATP production and oxidative phosphorylation, resulting in a higher AMP/ATP ratio which in turn inhibits gluconeogenesis and modulates AMPK. AMPK activation by metformin induces fatty acid oxidation, reduces lipid synthesis, and inhibits gluconeogenesis [63, 64]. AMPK is regulated by metformin via an upstream kinase, LKB1, which is produced by a tumour suppressor gene and controls cell growth. Although AMP-activated protein kinase is one of the most important molecular targets of metformin, this drug also has AMPK-independent effects on glucose metabolism, as it has been shown in mice that deficiency of LKB1 and AMPK following treatment with metformin leads to a reduction of serum glucose levels. These effects might be the result of the change in AMP/ATP ratio that regulates hepatic glucose output upstream of AMPK and suppresses cyclic-AMP-protein kinase A signaling [8]. Although the primary action of metformin is the metabolic homeostasis, its putative role in treating different types of cancer is under investigation [65, 66]. Currently, metformin effect in preventing a number of cancers has been demonstrated by many epidemiological and clinical data [37, 67, 68] but the molecular mechanisms are yet to be elucidated. The treatment of endometrial cancer cells with metformin leads to displacement of constitutively active K-Ras from the cell membrane, uncoupling the mitogen-activated protein kinase (MAPK) signaling pathway [69]. Additional anticancer activity could be its antiaromatase activity [38] which leads to a reduction in circulating estrogen levels in obese women and an upregulation of progesterone receptor expression by endometrial cancer cells [70]. Furthermore, metformin has antiangiogenic effects, directly scavenging free radicals and blocking endogenous reactive oxygen species [71]. A study* in vitro* has shown that it significantly reduces DNA damage and mutation rates [72]; this might be the explanation of the reduced risk of cancer that has been shown by many epidemiological studies through the use of metformin. The potential preventive and therapeutic role of this drug on breast cancer has also been studied. For instance, Jiralerspong et al. in a retrospective study of 2529 diabetic patients, including 68 on metformin and 87 not, showed that diabetic patients affected with breast cancer and treated with metformin had higher rates of complete response to neoadjuvant chemotherapy than those not taking metformin [73]. In cell culture, the proliferation of a wide range of cancer cells such as breast cancer cells is inhibited by metformin [74, 75] and Liu et al. reported the apoptotic effect of metformin in triple negative breast cancer cells [76]. Recently it has been shown that metformin may also target cancer-initiating cells. In particular, it was shown that its use in a subpopulation of breast cancer cells suppressed their growth and decreased the ability of these cells to form tumours in mice [77]; its combination with trastuzumab leads to a reduction of cancer-initiating cell population in Her2-amplified breast cancer cells [78]. Moreover, metformin can regulate breast cancer-initiating cell ontogeny through repression of the process of epithelial to mesenchymal transition (EMT) [79]. Overall, metformin benefits to breast cancer may be due to its role on cellular cycle and PI3K/Akt/mTOR signaling pathway and negative insulin effects on tumour development and growth.

### 3.2. Thiazolidinediones

Thiazolidinediones (TZDs) used in the treatment of metabolic diseases are a class of drugs also known as glitazones, including troglitazone, rosiglitazone, and pioglitazone [80]. Troglitazone was withdrawn from the worldwide market in 2000 due to an increased incidence of drug-induced hepatitis [81]. Glitazones are synthetic ligands containing a functional group in which thiazolidine serves as a dione and acts as an agonist of the peroxisome proliferator-activated receptor gamma (PPAR*γ*) whose endogenous ligands are free fatty acids (FFA) and eicosanoids [82]. Activated PPAR*γ* shapes a heterodimeric complex with retinoid X receptor (RXR) and binds to peroxisome proliferator hormone response elements, upregulating specific target genes and downregulating others [83]. Thiazolidinediones act as insulin-sensitizing agents, increasing fatty acid uptake and storage in adipose tissue, promoting adiponectin expression, and decreasing expression of 11*β*-hydroxysteroid dehydrogenase type 1 (11*β*-HSD) which converts cortisone to active cortisol [84]. PPAR*γ* can regulate the transcription and/or the activity of different key regulators of energy homeostasis where it can be considered as a control element of the cellular energy status [82]. Beneficial effects of these drugs are also studied in polycystic ovary syndrome showing that TZDs can reduce insulin resistance in PCOS women mainly acting on the adipocytes and the muscle cells [85, 86]. In addition to improving insulin resistance and compensatory hyperinsulinemia, administration of TZDs may increase the ovulation rate and pregnancy in patients affected with PCOS [87]. It has been observed that TZDs can modulate secretion of several endocrine hormones reducing androgen production or improving gonadotropins secretion [81]. Moreover, Hu et al. showed a weak PPAR*γ* mRNA expression in granulosa cells of PCOS patients, that varied in relation to the clinical features of the disease and was upregulated following administration of different dosages of insulin and/or rosiglitazone [88].* In vitro* experiments run on human ovarian cells demonstrate the negative regulation exerted by TZDs on steroidogenic enzymes 3-*β*-hydroxysteroid-dehydrogenase (3*β*-HSD) and aromatase [80, 89, 90].

Interestingly, as with metformin, glitazones can activate AMPK indirectly and regardless of PPAR*γ* involvement [91], through an increase of intracellular ADP : ATP ratio, which leads to the inhibition of respiratory chain and subsequent AMPK phosphorylation [62].

Considering the potential effects of glitazones on gynaecological tumorigenesis, Shah et al. showed that thiazolidinediones decrease vascular endothelial growth factor (VEGF) production by human luteinized granulosa cells* in vitro* [92]. On the contrary, another study, which characterized the response of ovarian xenograft tumours to the nonhypercalcemic vitamin-D2 derived anticancer agent (MT19c), observed a reduced efficacy of MT19c and cisplatin following stimulation of PPAR*γ* with rosiglitazone, suggesting that PPAR*γ* promotes survival for some ovarian tumour cells [93]. Overall, these lines of evidence—although limited and contrasting in some aspects—indicate that in some conditions these drugs may be considered as protective agents even if, due to their side effects, their use should come as a secondary option in the treatment of the metabolic problems linked to PCOS.

### 3.3. Inositols

**Figure 3:**
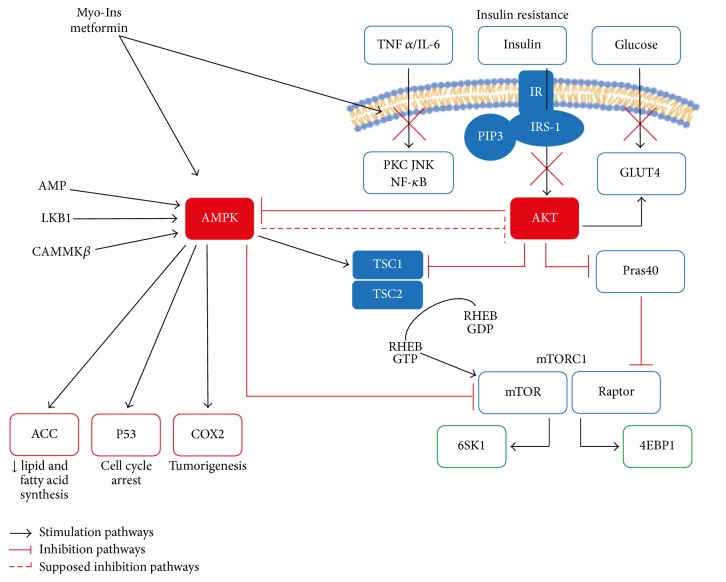
Negative regulation exerted by insulin-sensitizing on the molecular mechanism feeding insulin resistance. Role of AMPK as a metformin and myo-Ins molecular target. AMPK activation induces COX2 inhibition, p53 expression, and mTORC1 silencing through TSC2 and Raptor phosphorylation. The regulating activity of AMPK in processes of tumour induction and progression suggest that metformin may be proposed as an anticancer agent. TNF*α*: Tumour Necrosis Factor *α*; IL-6: interleukin 6; IR: insulin receptor; PKC: protein kinase C; JNK: c-Jun N-terminal Kinase; NF*κ*B: nuclear factor *κ*B; IRS-1: insulin receptor substrate 1; PI(3,4,5)P3: phosphatidylinositol 3,4,5-trisphosphate; Akt: protein kinase B; GLUT4: glucose transporter type 4; TSC1: tuberous sclerosis 1; TSC2 tuberous sclerosis 2; AMPK 5′: adenosine monophosphate-activated protein kinase; AMP: adenosine monophosphate; LKB1: liver kinase B1; CaMKK*β*: calcium/calmodulin-dependent protein kinase *β*; Pras40: Proline-Rich Akt/PKB Substrate 40 kDa; RHEB: Ras Homolog Enriched in Brain; mTOR: mammalian target of rapamycin; Raptor: regulatory-associated protein of mTOR; mTORC1: mammalian target of rapamycin complex 1; 6SK1: 6S Kinase 1; 4EBP1: Eukaryotic Translation Initiation Factor 4E-Binding Protein 1; ACC: Acetyl-CoA Carboxylase; p53: Tumour Protein 53; COX2: cyclooxygenase 2.

Inositol (cyclohexane-1,2,3,4,5,6) is a polyol of cyclohexane with six hydroxyl groups, identified for the first time in animal muscle tissue by J. J. Scherer in 1850. In the past it has been considered as a member of the vitamin B complex but inositol cannot be considered a “true” essential nutrient, as it can be synthesized by the human body. Indeed, cells can activate inositol biosynthesis, starting from glucose, through two enzymatic reactions. The first step consists of the conversion of D-glucose 6-phosphate to L-inositol-1P, catalyzed by 1L-myoinositol-1-phosphate synthase (MIPS). Subsequently L-Ins(1)P-phosphatase hydrolyzes L-inositol-1P forming myoinositol (myo-Ins) and orthophosphoric acid [94]. Inositols exist under nine stereoisomeric forms depending on the spatial orientation of their six hydroxyl groups. Myo-Ins and D-chiro-inositol (D-chiro-Ins) are the two main inositol stereoisomers naturally present in animal and plant cells, either in their free form or as bound-components of phospholipids or inositol phosphate derivatives. Myo-Ins is abundantly present in many plant sources and in certain high-fiber diets, such as cereals and legumes. When ingested, it is actively absorbed at the level of the gastrointestinal tract involving an Na^+^/K^+^-ATPasi [95]. Inositol transporters are involved in uptake and intracellular distribution of inositols that, according to their transport mechanism, can be classified as sodium ion coupled and proton coupled inositol transporters [96]. Myo-Ins is present in the cell embedded in the phospholipids docked to the plasma membrane and it is even a component of the glycosyl-phosphoinositides layered on the inner surface of the cellular membrane acting as important component of the calcium trafficking [97, 98]. The discovery of the second-messenger function of phosphatidylinositol, generated by the action of cytidine diphosphate diacylglycerol (CDP-DAG) inositol phosphatidyltransferase and its phosphorylated derivatives, the phosphoinositides, marked a turning point in studies of hormone function [99]. Myo-Ins plays an important role in the insulin signal transduction, lipid metabolism, calcium ions flow regulation, and assembly of cytoskeletal proteins. Recently, a substantial body of research evidenced its role in PCOS as an insulin-sensitizing agent affecting different pathways at both ovarian and nonovarian level [100]. In addition to being found in some food, myo-Ins is formed in the cells, its biosynthesis deriving from the conversion of D-glucose-6 phosphatase to L-inositol-1-phosphate [101]. Myo-Ins is an important component of the structural lipids and its various phosphates, including the phosphatidylinositol phosphate (PIP) lipids [102–104], are essential in maintaining the cellular membrane bilayer. Moreover, myo-Ins is the structural basis for many secondary messengers, including inositol triphosphates (InsP3), phosphatidylinositol (PI), polyphosphoinositides [i.e., PI(4)P, PI(4,5)P2, and PI(3,4,5)P3], and inositol phosphoglycans (IPGs), which controls several physiological events including regulation of hormone activities [105]. Inositol pyrophosphates act as a controlling factor of PI3K/Akt signaling pathway [106]. Myo-Ins is involved in mTORC1 and AMPK signaling activities [99]. Currently, myo-Ins is involved in inositol polyphosphate multikinase (IPMK) activity, a key enzyme for inositol polyphosphate biosynthesis and metformin-induced AMPK activation [107] (Figure 3).

In mammals, IPMK is also known as a physiologically important phosphatidylinositol 3 kinase (PI3K) that forms PI(3,4,5)P3 which activates Akt signaling pathway [108]. Recently, Kim et al. suggested the role of IPMK as a novel cofactor for mTORC1 signaling [109]. Moreover, IPMK also appears to be a novel AMPK-binding protein whose binding affinity for AMPK is dynamically controlled by glucose levels [107]. Myo-Ins intracellular homeostasis is adjusted by some processes, mainly the extracellular uptake via specialized myo-Ins transporters, phosphoinositide cycle, de novo biosynthesis from glucose-6-phosphate by 1-D-myoinositol-phosphate synthase (MIPS), and inositol monophosphatase (IMPase), efflux, and degradation [110]. Abnormalities in one or several of these processes lead to myo-Ins intracellular depletion found in conditions of hyperglycemia and insulin resistance as observed in diabetes mellitus [99]. Inositol transporters were identified in bacteria, protozoa, fungi, plants, and animals. According to their transport mechanisms, they can be classified into two groups: sodium-dependent myo-inositol transporters 1 and 2 (SMIT1/2) and proton coupled inositol transporters (HMIT) [105]. Many lines of evidence support the benefits of myo-Ins supplementation for some metabolic disorders associated with insulin resistance, because of its insulin mimetic properties. As mentioned above, PCOS is characterized by metabolic features such as central obesity and insulin resistance with compensatory hyperinsulinemia that also are key factors in the pathogenesis of chronic anovulation [111–113]. Myo-Ins, through its insulin-sensitizing effect, plays an important role in improving metabolic and hormonal parameters in women affected with PCOS [114, 115]. However, the best therapy for this disorder is constituted by the treatment with myo-Ins plus D-chiro-Ins combined in agreement with the physiological myo-Ins/D-chiro-Ins plasma ratio (40 : 1) [116–119], which allows obtaining very interesting results and also avoids some detrimental effects due to D-chiro-Ins alone at high concentrations [120]. Myo-Ins involvement in the reproductive axis function is highlighted by the pivotal role played by inositol (1,4,5)-triphosphate [Ins(1,4,5)P3] in the regulation of calcium ion (Ca^2+^) release during oocytes development and its role in meiotic competence and the final stage of oocyte maturation [121]. In PCOS patients, a defect in tissue availability or an altered metabolism of inositol or IPGs mediators may contribute to insulin resistance [122, 123]. As myo-Ins involvement in PI3K/Akt/mTOR and AMPK signaling pathway [124] plays important roles in the regulation of cellular growth and survival it is interesting to examine its possible role in cancer development. Han et al. have shown that regression of bronchial dysplastic lesions can be caused by myo-Ins through inhibition of active Akt and ERK and its molecular target,* in vivo* and* in vitro* [125]. Furthermore, a phase 1 clinical study carried out in order to determine the potential chemopreventive effect of myo-Ins in smokers with bronchial dysplasia showed a potential positive effect at the oral daily dose of 18 gr for three months [126] without significant adverse events despite the high doses used. Indeed, myo-Ins may work through multiple mechanisms to inhibit tumour progression. Interest is growing for inositol hexaphosphate (InsP6), a natural occurring polyphosphorylated carbohydrate contained in high-fiber diets and present in mammalian cells, and for its role in cancer prevention as well as control of experimental tumour growth, progression, and metastasis [127]. Dong et al. reported that InsP6 strongly inhibited cellular transformation induced by epidermal growth factor (EGF), ERK, and PI3K activation [128]. Based on the synergic activity of myo-Ins and metformin in metabolic disorders characterized by insulin resistance, and according to the suggested antitumoural effect of metformin treatment, the possibility that myo-Ins might have some ability to inhibit tumour growth also in gynaecological cancer should be considered. More studies are needed to clarify a possible anticancer role of myo-Ins in patients affected with PCOS.

## 4. Insulin-Sensitizers in PCOS and Ovarian Cancer

Ovarian cancer (OC) is the eighth most common cancer and the seventh most common cause of death from cancer in women worldwide. There are factors that increase the risk to develop OC but they are still not well validated. The onset of OC can be caused by many factors such as age, family history of OC, infertility treatment and assisted fertilization, hormonal substitution in menopause, and obesity [129]. Endocrine disorder associated with hypersecretion of ovarian androgens, anovulation, and menstrual irregularity in PCOS seem to be the cause of higher risk of the epithelial ovarian cancer development due to the abnormal hormonal environment. In fact, in PCOS, we see hormonal alteration with abnormal concentrations of unopposed oestrogens. Several hypotheses are made to explain ovarian carcinogenesis. The majority of malignant ovarian tumours seem to have steroid hormone receptors (62% for oestrogen, 49% progesterone, and 69% for androgen) [130]. Berchuck et al. proposed that an interference in the local concentration of growth factors and steroids conducts malignant changes in the ovarian epithelium [131]. Oestrogens prevent apoptosis through Bcl-2 upregulation and this may lead to ovarian carcinogenesis, whereas progesterone has a proapoptotic effect on ovarian epithelium through either the modulation of transforming growth factor *β* (TGF *β*) isoform expression or the activation of the Fas/Fas Ligand signaling pathway [132]. Also androgens seem to be involved in ovarian carcinogenesis in animal models and testosterone may intensify the ovarian epithelial tumours growth [133]. Androgens decrease TGF *β* receptor levels, allowing tumour cells to escape TGF*β*1 mediated growth inhibition, thus promoting ovarian cancer progression [134]. Repeated anovulatory cycles, with the generation of inclusion cysts, may increase the risk of genetic damage, because of exposure of the epithelial cells to high concentrations of oestrogens in follicular fluid [135]. Likewise, high levels of circulating gonadotropins increase the risk of developing ovarian cancer, especially in early postmenopausal years [136]. In the literature, few and conflicting studies addressing the possibility of an association between ovarian cancer and PCOS are reported [137], with the additional problem raising from uncertainties in PCOS diagnostic criteria, as most of such studies have been conducted before the Rotterdam ESHRE/ASRM PCOS Consensus 2003 [138]. In 1996, Schildkraut et al. demonstrated in a population-based case-control study a 2.5-fold increase in the risk of ovarian cancer among women with PCOS [139]. A stratified analysis adjusted for age found that oral contraceptive use plays a protective role in women with PCOS [139]. Stratified age-adjusted analysis also showed that younger women reporting to have PCOS were at much greater risk for developing ovarian cancer [139]. However, the small number of women with PCOS (*n* = 31) limited the interpretation of these findings, with the possibility of recall bias in subjects affected with ovarian cancer [140]. An Australia-wide population-based case-control study of subjects aged 18–79 years with new diagnosis of invasive epithelial ovarian cancer (*n* = 315) versus controls (*n* = 1508) tested the hypothesis that hyperandrogenism is associated with the genesis of ovarian cancer. The authors found no evidence that self-reported histories of PCOS were associated with an increased ovarian cancer risk, although women with PCOS who were also overweight had a significantly increased risk of serious borderline tumours [141]. A case-control analysis of 1611 patients versus 9170 controls with a diagnosis of ovarian cancer shows that PCOS (OR 1.63 95% CI 0.65–4.08) was associated with a tendency towards an increased risk of ovarian cancer. A retrospective case-control analysis has shown that long-term use of metformin was associated with lower rates of ovarian cancer (OR 0.61, 95% CI 0.3–1.25) [142]. Identification of a number of proteins overexpressed in both PCOS and ovarian cancer, such as superoxide dismutase, calreticulin, vimentin, fibrinogen *γ*, lamin B2, and malate dehydrogenase, assured the identification of women with PCOS with an increased risk of developing this malignancy [143]. A systematic review and meta-analysis of observational studies considered for PCOS association and ovarian cancer only three studies [144]. The OR resulted significantly higher in the single study of women aged <54 years [139]; however the increased risk of ovarian cancer in women with PCOS was not significant [144]. This is in line with a nationwide population-based retrospective cohort study, conducted in Taiwan, where also the increased risk of ovarian cancer in the PCOS group was not reported [145]. The analysis of a large cohort study from the Danish National Patient Register compared the women's cancer incidence with that of the general Danish female population by means of standardized incidence ratios (SIRs) and found no association between PCOS and ovarian cancer [146]. The association with obesity is not pronounced as much as the association with endometrial cancer; however a recent meta-analysis and a systematic review found that ovarian cancer was barely more common in women with a BMI ≥ 30 kg/m^2^ [147, 148] and a prospective cohort study showed increased mortality from ovarian cancer among overweight and obese women compared with normal weight women [149]. Lee at al. reported a higher risk of ovarian cancer in diabetic women, which persisted after adjusting for BMI, age, alcohol intake, and smoking [148]. Numerous prospective observational studies suggested that not only patients with type 2 diabetes taking metformin were at lower risk of developing cancer [150] but also mortality was less common [151]. Stadtmauer et al. suggested that insulin-sensitizing agents use, as well as improving reproductive function, produces long-term health benefits [152]. Also myo-Ins acts as a controlling factor of PI3K/Akt signaling pathway [99] and it is also involved in mTORC1 and AMPK signaling [107]. Thus, besides its insulin-sensitizing effect, it plays an important role in improving metabolic and hormonal parameters in women affected with PCOS and a possible chemopreventive effect can be hypothesized. The identification of a high risk group is important, and this may include women with PCOS, impaired glucose tolerance, morbid obesity, endometrial hyperplasia, and risk for developing ovarian cancer.

## 5. Insulin-Sensitizers in PCOS and Endometrial Cancer

A growing scientific interest towards addressing the risk of endometrial cancer in women affected by polycystic ovarian syndrome is emerging in endocrinology and gynaecologic oncology. Currently, endometrial cancer (EC) is the most common gynaecological malignancy in Europe and North America, with a global tendency to further increase over time, mainly involving postmenopausal women [153]. The main risk factors regarding hormone-related cancer are recognized in exposure to unopposed estrogen therapy, overweight and obesity, late-age menopause, and nulliparity [114, 115] and therapy with Tamoxifen as well [154]. Since EC risk is strongly associated with high circulating oestrogen levels, all of the pathological conditions leading to this hormonal status may promote endometrial proliferation. This is the case of women affected by PCOS that show a high risk of developing atypical endometrial hyperplasia (EH), which is considered a precancerous lesion, which may progress to EC. An assessment of the prevalence of EH in PCOS women still remains a widely debated issue. Although the hyperplastic rate has been reported as high as over 48% [155], others [156] did not confirm it, referring to an overall prevalence close to 1%. As already mentioned [157], such a wide range of EH prevalence in PCOS patients may reflect the heterogeneous nature of the PCOS phenotypes and the varying diagnostic criteria over time. The biological relationship between PCOS and EC, although still unclear, refers to a combination of complex reproductive and metabolic disorders: likewise, chronic anovulation, obesity, and hyperinsulinemia, resulting in progesterone deficiency. Consequently, the endometrium tends to remain in an oestrogen dominant proliferative state, increasing the risk of developing uterine cancer [158, 159]. Furthermore, metabolic impairment in PCOS consists in insulin resistance accompanied by an increase of IGF-I, both playing a pivotal role in the endometrial cell proliferation and differentiation, promoting the development of EC [160, 161] as follows.

*Speculated Pathogenesis of Endometrial Cancer in PCOS Women (Mod from Gadducci et al. [132])*

 Chronic anovulation → unopposed estrogen stimulation
   ↑ serum LH
   ↑ endometrial LH receptor expression
 Insulin resistance → chronic hyperinsulinemia
   ↑ serum IGF-1

The estimated risk of PCOS women in developing endometrial cancer has been evaluated in some observational case-series, case-control studies, systematic reviews, and meta-analysis as well. With only a few exceptions [162], the majority of such studies [144, 159, 163–166] confirmed a higher EC risk for PCOS women. In the early 90s, one of the first case-control studies [167] managed by the National Cancer Institute program of the Surveillance Epidemiology and End Result, assigned to PCOS women an odd ratio (OR) for endometrial cancer of 2.7 compared to the control group. In a further retrospective study, Wild et al. [163] evaluated the long-term endometrial consequences in PCOS women reporting, over 30 years of follow-up, a higher OR (up to 5.3) for developing EC. A systematic review and a meta-analysis by Chittenden et al. [166] explored the long-term outcome of PCOS patients. Among four observational studies, they reported that women with PCOS appear to be three times more likely to develop well-differentiated endometrial cancer. More recently Haoula et al. [159] performed a meta-analysis comparing previous observational studies and confirmed that the risk of developing EC is three times more likely compared with general population. Furthermore, Barry et al. [144] in a case-control study have reported a significantly increased OR of 2.79 for endometrial cancer in women with PCOS and endometrial hyperplasia. Moreover when the meta-analysis was restricted to women younger than 54 years, the risk estimate further increased (OR of 4.05). In a large Danish cohort study, using the National Patient and Cancer Registry [146], during the period 1977–2012, the incidence of endometrial cancer was significantly higher in PCOS-affected women less than 50 years of age, with a standardized incidence ratio of 3.9. Most of these cases were type I endometrial cancer, a finding that supports the pathogenetic mechanism of long-term exposure to unopposed oestrogen.

Finally, a high-scale nationwide population-based cohort study, conducted in Taiwan [145], retrospectively estimated the risk for EC of 8.42 times higher for women with PCOS than for the comparison cohort. All these lines of evidence suggest, as stated by the 3rd PCOS Consensus Group [168], that there are moderate-quality data supporting the high risk of PCOS women for developing endometrial cancer, with consequent need for increased awareness for surveillance strategies. As metformin increases signaling by the insulin receptor, leading to an improvement in insulin resistance and a reduction in circulating insulin levels, it is regularly used as an insulin sensitizer. Furthermore, metformin plays an essential role in the inhibition of hepatic gluconeogenesis [169] with the consequent decrease in insulin levels. Thus, metformin is a cornerstone in treatment of PCOS women for improving reproductive abnormalities, restoring ovulation, and improving fertility [170, 171]. The recognition of PCOS as a risk factor for endometrial cancer has some, although still debated, therapeutic implications. Progesterone and its analogues, as well as a levonorgestrel-releasing intrauterine device, are effectively used to inhibit oestrogen-induced endometrial proliferation while promoting synchronous growth, development, and shedding of a structurally stable endometrium. However, it has been documented that up to 30% of PCOS women with endometrial hyperplasia do not correctly respond to this type of hormonal approach [172, 173], possibly due to a progesterone-resistance, which in turn determines persistence of endometrial hyperplasia and further possible cancer transformation. Such hormonal-resistance may be defined as the clinical failure after high-dose progesterone treatment for 3 months, resulting in the acceleration of atypical EH [14]. To overcome this resistance, metformin therapy may be used in PCOS women affected by proliferative endometrial pathologies. Glucose metabolism in PCOS women and reversing endometrial proliferation as well as modulation of insulin sensitivity are functions of metformin very commonly described. Indeed, an endometrial cancer model has described that exposure of EC cells to sera from PCOS under metformin therapy reduces cell growth, altering signaling pathways involving tumour invasion [14]. Thus, a pivotal biological pathway leading to endometrial cancer may be theoretically controlled by metformin, to reduce the risk of EH progression [174]. Given this background, metformin therapy, with/without progesterone-based oral contraceptives, has been adopted in small anecdotal studies to reverse atypical EH or early-stage EC as well (Table 1). In an early case-report, following the failure of progestogen therapy, Session et al. [10] reported EH regression after metformin. Some year later, Shen et al. [12] confirmed metformin and progestin-based contraceptives as being effective therapeutic combination in two women affected by atypical EH with progestin-resistance.

However, in regard to endometrial cancer outcomes, data concerning a protective efficacy of metformin are still insufficient [157]. In a large retrospective cohort study [175], the use of metformin among diabetic patients with nonendometrioid EC was associated with a statistically improved overall survival (OS). Similarly, in another retrospective cohort analysis EC diabetic patients using metformin had an improved recurrence-free survival and OS than those who did not use metformin [176]. Conversely, other results from observational case-control analysis [177] did not confirm a decreased risk of endometrial cancer in metformin users. In addition to this, due to the lack of studies, a systematic review carried out using the Cochrane Library database [178] did not reach any firm conclusion about a preventive role of metformin in endometrial cancer. Some data support that metformin treatment, for 6 months, results in decreased incidence, progression, and even cancer-related mortality with regard to endometrial cancer. Li et al. [15] described the efficacy of a combination strategy with hormonal therapy (cyproterone acetate and ethinyl estradiol, Diane) and metformin in five PCOS women affected by stage IA endometrial cancer. After 6 months, all of the women reverted to normal endometrium. Even though it was suggested that metformin might become an important pharmacological asset in early-stage endometrial cancer [54], further prospective investigations are needed. For this reason, the North Carolina Lineberger Comprehensive Cancer Center started a pilot study to evaluate the effectiveness of metformin in reverse endometrial hyperplasia. Currently the study is still ongoing, recruiting participants, with an estimated study completion date in December 2018 [179]. Furthermore, at the same Cancer Center, another study is in progress to assess the conservative treatment of EH, with oral progestin or levonorgestrel-intrauterine device (LNG-IUS) plus metformin therapy [180].

## 6. Conclusions

Several preclinical cancer models, including studies on endometrial cancers cell lines, have shown that insulin-sensitizers might act as anticancer agents in ovarian and endometrial cancers and their ability to act on a variety of pathways, with wide-ranging effects, should be taken into consideration. Although a lot of evidences support the potential role of insulin-sensitizers in prevention and treatment of certain gynaecological cancer, further research efforts are required to confirm their clinical usefulness. In fact, it has been demonstrated that they activate AMPK, a potent inhibitor of the PI3K/Akt/mTOR pathway, prompt G1 cell cycle arrest, induce apoptosis, and decrease human telomerase reverse transcriptase expression. Furthermore, they seem to inhibit mTOR through AMPK-independent pathways, which interfere with the development and DNA damage response mechanisms. Insulin-sensitizers antiangiogenetic effects reduce DNA damage and mutation rates offering an explanation for the reduced risk of cancer seen in metformin users across several epidemiological studies. PCOS seems to be associated with an increased risk for developing epithelial ovarian cancer due to the abnormal hormonal environment with abnormal concentrations of unopposed oestrogen, even if few studies support the fact that the association between them is limited. Androgens such as testosterone can enhance the growth of ovarian epithelial tumours in animal models and may promote ovarian cancer progression by decreasing TGF*β* receptor levels, thereby allowing tumour cells to escape TGF*β* 1 mediated growth inhibition. Recently a number of proteins overexpressed in both PCOS and ovarian cancer, such as superoxide dismutase, fibrinogen *γ*, vimentin, calreticulin, malate dehydrogenase, and lamin B2, have been identified, revealing subgroups of women with PCOS and with an increased risk of developing this malignancy. Metformin appears to decrease endometrial cancer incidence, progression, and even mortality, in particular at an early stage. It appears reasonable to assume that insulin-sensitizers drugs, by reducing the carcinogenic effects of obesity and insulin resistance, might be employed for long-term chemoprevention in women at high risk of endometrial cancer. It is necessary to carry out new retrospective epidemiological research and meta-analysis on wide population of PCOS patients organized in groups based on reproductive axis function, hormonal and metabolic profile, and drugs therapies. Therefore, even though tumoural cell cultures and mouse models have been used to explain insulin-sensitizers' mechanism of action and their potential inhibitory effect on tumourigenesis, new and more physiologically relevant* in vitro* human models are needed to fully elucidate the molecular mechanisms exploited by these drugs and shape clinical studies. Additional research would be useful to better define cytokines and growth factors that are activated in gynaecological cancers and may modulate cellular responses to insulin-sensitizers in women suffering from PCOS. This is an important key point in identifying the most suitable patients to be treated with these drugs and plan adequate clinical trials.

## Figures and Tables

**Table 1:** Metformin and endometrial proliferative pathology.

Author	Ref	Year	Study	Results
Session et al.	[10]	2003	Human clinical study	MTF regresses atypical endometrial hyperplasia in one patient
Legro et al.	[11]	2007	Human clinical study	MTF resolved simple hyperplasia in two patients
Shen et al.	[12]	2008	Human clinical study	MTF regresses atypical endometrial hyperplasia in two women
Cantrell et al.	[13]	2010	Endometrial cell line experimental	MTF is a potent inhibitor of cell proliferation in endometrial cancer cell lines
Tan et al.	[14]	2011	Human experimental study	MTF reduces *in vitro* endometrial invasion
Li et al.	[15]	2014	Human clinical study	MTF reverts stage IA endometrial carcinoma into normal in five young PCOS patients

MTF: metformin.

## Abbreviations

ACC: Acetyl-CoA Carboxylase
Akt/PKB: Protein kinase B
AMP: Adenosine monophosphate
AMPK: 5′ Adenosine monophosphate-activated protein kinase
ATP: Adenosine-5′-triphosphate
BAD: Bcl2-Antagonist of cell Death
Ca^2+^: Calcium ions
CDP-DAG: Cytidine diphosphate diacylglycerol
CaMKK: Calcium/calmodulin-dependent protein kinase
COX-2: Cyclooxygenase 2
CYP17: Cytochrome P-45017
DAG: Diacylglycerol
D-chiro-Ins: D-Chiro-inositol
EC: Endometrial cancer
EGF: Epidermal growth factor
EH: Endometrial hyperplasia
EMT: Epithelial to mesenchymal transition
ERK: Extracellular-signaling regulated protein kinase
4E-BP1: Eukaryotic Translation Initiation Factor 4E-Binding Protein 1
FFA: Free fatty acids
GLUT: Glucose transporter
GPCR: G-protein coupled receptor
GSK3*β*: Glycogen synthase kinase 3
HIF: Hypoxia inducible factor
HMIT: Proton coupled inositol transporters
11*β*-HSD: 11*β*-Hydroxysteroid dehydrogenase
3*β*-HSD: 3*β*-Hydroxysteroid-dehydrogenase
IGF-I: Insulin growth factor 1
IGF-II: Insulin growth factor 2
IL-6: Interleukin 6
IR: Insulin receptor
Ins(1,4,5)P3: Inositol (1,4,5)-triphosphate
InsP6: Inositol hexaphosphate
IPMK: Inositol polyphosphate multikinase
IRSs: Insulin receptor substrates
ISF-1: Insulin sensitivity factor 1
JNK: c-Jun N-terminal Kinase
K-Ras V-Ki-ras2: Kirsten rat sarcoma viral oncogene homolog
LH: Luteinizing hormone
LKB1: Liver kinase 1
MAPK: Mitogen active proteins kinase
MIPS: 1L-Myoinositol-1-phosphate synthase
MO25: Mouse protein 25
myo-Ins: Myoinositol
MT19c: Nonhypercalcemic vitamin-D2 derived anticancer agent
mTOR: Mammalian target of rapamycin
mTORC1: Mammalian target of rapamycin complex 1
MTF: Metformin
NF-*κ*B: Nuclear factor *κ*B
OCT1: Organic Cation Transporter 1
OR: Odd ratio
p53: Tumour Protein 53
PCOS: Polycystic ovary syndrome
PDK1: Phosphoinositide dependent protein kinase-1
PI: Phosphatidylinositol
PI(4,5)P2: Phosphatidylinositol 4,5-biphosphate
PI(3,4,5)P3: Phosphatidylinositol 3,4,5-trisphosphate
PI3K: Phosphatidylinositol-3-kinase
PIP: Phosphatidylinositol phosphate
PKC: Protein kinase C
PLC*γ*: Phospholipase C *γ*
PPAR*γ*: Peroxisome proliferator-activated receptor gamma
PRAS40: Proline-Rich Akt/PKB Substrate 40 kDa
PTEN: Phosphatase and tensin homolog
RAC1: Ras-Related C3 Botulinum Toxin Substrate 1
REDD1: Regulated in development and DNA damage responses 1
RHEB: Ras Homolog Enriched in Brain
RPTOR: Regulatory-associated protein of mTOR
RR: Relative risk
RXR: Retinoid X receptor
S6K1: Ribosomal protein S6 kinase 1
TAK1: Transforming growth factor *β*-activated kinase 1
SHBG: Sex hormone binding globulin
SMIT1/2: Sodium-dependent myoinositol transporters 1 and 2
STRAD: STe20-Related Adaptor
TGF*β*: Transforming growth factor *β*
TNF*α*: Tumour Necrosis Factor *α*
TSC2: Tuberous sclerosis complex-2
TZDs: Thiazolidinediones
VEGF: Vascular endothelial growth factor.
